# Prediction of induction chemotherapy efficacy in patients with locally advanced nasopharyngeal carcinoma using habitat subregions derived from multi-modal MRI radiomics

**DOI:** 10.3389/fonc.2025.1539574

**Published:** 2025-05-14

**Authors:** Mulan Pan, Lu Lu, Xingyu Mu, Guanqiao Jin

**Affiliations:** ^1^ Department of Radiology, Guangxi Medical University Cancer Hospital, Guangxi Clinical Research Center for Imaging Medicine, Guangxi Clinical Key Specialty (Medical Imaging), Key Discipline Development Program (Medical Imaging), Affiliated Cancer Hospital of Guangxi Medical University, Nanning, China; ^2^ Department of Nuclear Medicine, Guangxi Academy of Medical Sciences, People’s Hospital of Guangxi Zhuang Autonomous Region, Nanning, China; ^3^ Department of Nuclear Medicine, Affiliated Hospital of Guilin Medical University, Guilin, China

**Keywords:** nasopharyngeal carcinoma, habitat subregion, radiomics, machine learning, RF

## Abstract

**Objective:**

This study aims to predict the early efficacy of induction chemotherapy (ICT) in patients with locally advanced nasopharyngeal carcinoma (LA-NPC) through habitat subregion analysis and multimodal MRI radiomics techniques.

**Methods:**

The study employed a retrospective design and included LA-NPC patients who received ICT treatment between 2015 and 2019. The K-means clustering algorithm was utilized to segment the tumor into five distinct habitat subregions based on imaging features. A total of 2,153 radiomic features, including geometric shape, intensity, and texture features, were extracted. Feature selection was conducted using the maximum relevance minimum redundancy (mRMR) method and the least absolute shrinkage and selection operator (LASSO) technique. Eleven machine learning algorithms were employed to develop radiomics models based on the CE-T1WI and T2-FS sequences, respectively. These models were evaluated using various predictive performance metrics, including area under the curve (AUC), sensitivity, and specificity. Model selection was based on comprehensive cross-validation performance and AUC values.

**Results:**

The study population comprised 76.63% males and 23.37% females, with a mean age of 42.60 ± 10.21 years. All patients had stage III to IVa nasopharyngeal carcinoma, and the majority (92.39%) had non-keratinizing squamous cell carcinoma. Habitat subregion analysis revealed that the volume features of a specific subregion (Subregion 2) were significantly associated with patient response to ICT (*P* = 0.032). The RF model built using radiomic features from Subregion 2 demonstrated the best performance on the CE-T1WI sequence, with an AUC of 0.921 in the training set and 0.819 in the testing set. On the T2-FS sequence, the Random Forest (RF) model also exhibited high diagnostic performance, with an AUC of 0.933 in the training set and 0.829 in the testing set. These results suggest that the RF model provides stable and reliable predictive performance across different MRI sequences.

**Conclusion:**

Habitat subregion analysis using multimodal MRI radiomics offers an effective approach for the early identification of LA-NPC patients with poor responses to induction chemotherapy. This method holds promise for supporting clinical treatment decisions and achieving personalized medicine.

## 1 Introduction

Nasopharyngeal carcinoma (NPC) is an aggressive cancer that originates from the delicate mucosa in the upper throat, located behind the nose. This type of cancer is notably prevalent in specific geographic regions, particularly in Southeast Asia and southern China, where it has become a significant public health concern. In these regions, the most prevalent subtype of NPC is non-keratinizing squamous cell carcinoma, accounting for over 95% of all cases. This highlights the disease’s unique pathological characteristics in these populations (1, 2). Furthermore, NPC exhibits a pronounced regional clustering, with more than 75% of the global incidence of this malignancy reported in Southeast Asia and southern China, indicating a strong correlation between geographical factors and the prevalence of this cancer (3, 4). The tumor’s unique location and the subtle, nonspecific early symptoms often lead to a diagnosis in 70% of patients at an advanced stage. This complicates treatment options and worsens prognosis (5).

For patients suffering from locally advanced nasopharyngeal carcinoma (LA-NPC), recent studies have shown that the combination of induction chemotherapy (ICT) followed by concurrent chemoradiotherapy significantly improves prognosis when compared to chemoradiotherapy administered alone (6). However, it is important to note that not all patients derive the same level of benefit from ICT. Research, including a pivotal study by Liu, shows that about 23% of NPC patients do not respond well to ICT, highlighting the need for personalized treatment strategies (7). Therefore, identifying sensitivity to ICT prior to the initiation of treatment is critical for the development of personalized therapeutic strategies that can enhance patient outcomes.

To address this challenge, researchers are using specific quantitative parameters derived from functional MRI as predictive tools to evaluate the effectiveness of chemoradiotherapy in patients with LA-NPC (8, 9). However, these methodologies often face limitations, as they tend to focus on analyzing specific regions of interest (ROIs) within the tumor, thereby neglecting a comprehensive assessment of the entire tumor and failing to capture the full extent of intratumoral heterogeneity that may influence treatment response. Additionally, functional MRI itself presents several drawbacks, including prolonged scan times, high costs associated with the technology, and the complex requirements for post-processing the acquired imaging data.

Given these challenges, radiomics has emerged as a promising technology that extracts high-dimensional features from medical images. This process transforms these features into quantitative data, providing deeper insights into tumor characteristics (10–12). This innovative approach has demonstrated significant potential for identifying imaging biomarkers that can aid in the assessment of tumors. However, traditional radiomics analyses typically assume tumors are homogeneous. This assumption overlooks the phenotypic heterogeneity present in different tumor regions, limiting the effectiveness of the technique in capturing the full complexity of nasopharyngeal carcinoma (13).

To address this limitation, a novel methodology known as habitat subregion analysis has been introduced into the field of oncology. This approach divides tumors into distinct subregions, each containing voxel clusters that show similar imaging features. This effectively characterizes and quantifies the complex intratumoral heterogeneity that can significantly influence treatment outcomes (14–16). Numerous studies have shown that habitat subregion analysis has strong prognostic capabilities across various cancers, such as breast, lung, and brain cancers (17–21). This highlights its potential as a valuable tool in cancer diagnostics and treatment planning. However, current predictive tools based on functional MRI face challenges in capturing tumor heterogeneity.

Although some research has explored habitat subregion analysis in NPC, its utility for predicting early responses to ICT in LA-NPC has not been thoroughly investigated and requires further study (22, 23). This study is designed with the objective of developing and validating a comprehensive habitat subregion radiomics model that leverages multimodal MRI data to accurately predict the efficacy of early ICT in patients diagnosed with LA-NPC. By achieving this goal, we aspire to facilitate personalized treatment planning that is tailored to the unique characteristics of each patient’s tumor, ultimately aiming to enhance survival outcomes and improve the quality of life for individuals battling this challenging disease.

## 2 Materials and methods

### 2.1 Study population

This retrospective, single-center study included 265 patients with LA-NPC who received ICT at the Affiliated Cancer Hospital of Guangxi Medical University from January 2015 to June 2019. The inclusion criteria were as follows: (1) histologically confirmed NPC (stages III–IVa, as per the 8th AJCC staging system); (2) Karnofsky Performance Status (KPS) of 70 or higher and Eastern Cooperative Oncology Group (ECOG) score of 0–1; (3) age between 18 and 70; (4) eligibility for both plain and contrast-enhanced MRI; (5) imaging confirming the extent of primary lesions and lymph node metastases; (6) normal liver and kidney function, no severe hematological toxicity, and no major medical comorbidities; (7) no previous surgical interventions for NPC; and (8) voluntary written informed consent. The exclusion criteria included: (1) age over 70 years; (2) KPS below 70 or ECOG score above 1; (3) ineligibility for ICT or concurrent chemoradiotherapy due to medical contraindications; (4) prior radiotherapy to the head and neck; (5) presence of metal implants (e.g., cardiac pacemakers, orthopedic fixation devices), dentures, or prosthetics that may cause MRI artifacts, which could significantly degrade image quality and compromise tumor delineation; and (6) claustrophobia.

### 2.2 Chemotherapy protocol

Patients received two cycles of ICT comprising: (1) Docetaxel (70 mg/m², based on TAX323/TAX324 trial protocols with dose modification for tolerability) or albumin-bound paclitaxel (260 mg/m², per NCCN guideline recommendations for head and neck cancers) on Day 1; and (2) Cisplatin (40 mg/m² on Days 1 and 2, adapted from the split-dose regimen in the DECIDE trial to mitigate renal toxicity while maintaining cumulative dose intensity of 80 mg/m² per cycle) (24, 25). Dosage adjustments were permitted based on individual renal function, body surface area calculation, and treatment-related toxicity monitoring.

### 2.3 Efficacy evaluation

In therapeutic efficacy evaluation for patients undergoing ICT, two board-certified radiation oncologists with >10 years’ clinical experience performed short-term treatment response evaluations following Response Evaluation Criteria in Solid Tumors (RECIST) version 1.1 (26). The assessment incorporated multimodal clinical data encompassing imaging studies, nasopharyngoscopy results, and presenting symptoms. Discrepancies between evaluators were resolved through consensus discussion. Treatment outcomes were stratified into four distinct categories: Complete Response (CR), characterized by total resolution of all target lesions; Partial Response (PR), defined as ≥30% reduction in the sum of target lesion diameters from baseline; Progressive Disease (PD), indicating ≥20% increase in lesion diameter sums relative to nadir measurements; and Stable Disease (SD), representing disease states fulfilling neither PR nor PD criteria. For comparative analysis, CR cases comprised the complete response cohort, whereas PR, SD, and PD cases were consolidated into the non-CR group. All MRI analyses were conducted by certified radiologists using standardized protocols to ensure interpretive consistency. This stratification system enables systematic investigation of therapeutic response heterogeneity and prognostic implications.

### 2.4 MRI scanning protocol

MRI scans were performed within three days before the ICT and again after the second ICT cycle, which took place 21 to 24 days later. A 1.5T Siemens MAGNETOM AVANTO or a 3.0T GE DISCOVERY MR750 with a 16-channel head and neck coil was utilized. The imaging sequences included diffusion-weighted imaging (DWI), diffusion kurtosis imaging (DKI), intravoxel incoherent motion (IVIM), and dynamic contrast-enhanced MRI (DCE-MRI). Additionally, contrast-enhanced T1-weighted imaging (CE-T1WI) and T2-weighted imaging (T2-FS) were exported for analysis. MRI parameters were as follows: for the 1.5T MRI, CE-T1WI (TR/TE = 885 ms/19 ms; matrix = 256 × 256) and T2-FS (TR/TE = 6760 ms/91 ms; matrix = 768 × 696); for the 3.0T MRI, CE-T1WI (TR/TE = 4748 ms/65 ms) and T2-FS (TR/TE = 682 ms/10 ms). The slice thickness was 5 mm, the interslice gap was 1 mm, and the field of view (FOV) was 374 mm × 240 mm. Contrast enhancement was achieved using gadoterate meglumine at a dosage of 0.2 mL/kg.

### 2.5 MRI image preprocessing and lesion segmentation

Before delineating the volume of interest (VOI), we preprocessed the MRI images with ITK-SNAP software (version 3.8.0, http://www.itksnap.org), which involved voxel resampling, bias correction for MRI field strength, and Gaussian denoising. Voxel resampling standardized all images to a voxel size of 1 × 1 × 1 mm³ to achieve isotropy, thereby maintaining rotational invariance during texture feature computation. The primary lesions of NPC observed on T2-FS and CE-T1WI images were used as the basis for delineating the VOI. A junior radiologist with five years of experience in head and neck cancer diagnosis performed three-dimensional manual segmentation of the VOI using ITK-SNAP software. The VOI, delineated based on the NPC primary lesions, encompassed the entire tumor across each contiguous slice of T2-FS and CE-T1WI images while avoiding cystic and necrotic areas (focal high-signal areas on T2-FS or low-signal areas on CE-T1WI). Subsequently, a senior radiologist with 25 years of experience in head and neck cancer diagnosis randomly selected T2-FS and CE-T1WI images from 30 cases and redelineated the VOI using the same method and software to assess inter-observer consistency by calculating the intraclass correlation coefficient (ICC). Local features of voxels from the entire tumor region of interest were extracted using tools from the “Onekey” platform (OKT-gen-roi-rad-features), and the K-means unsupervised algorithm was employed for clustering analysis of all samples, with clustering effectiveness typically evaluated using inter-cluster and intra-cluster distances.

(1) The Calinski-Harabasz score, also known as the variance ratio criterion, is used to measure the cohesion and separation of clusters. This score evaluates clustering quality by comparing within-cluster variance and between-cluster variance. The calculation formula is as follows:


$$CH=\frac{Between-cluster\;dispersion}{within-cluster\;dispersion}\times \frac{n-k}{k-1}$$

Where (n) is the total number of samples, (k) is the number of clusters, the between-cluster variance indicates the degree of separation between different clusters, and the within-cluster variance reflects the tightness within clusters. A higher CH value indicates greater between-cluster differences, higher within-cluster tightness, and better clustering performance.

(2) The Silhouette Coefficient is a metric that measures the similarity of clustering results, taking into account the similarity of each sample to other samples in its cluster as well as the similarity to the nearest cluster. The calculation formula is as follows:


$$s\left(i\right)=\frac{b\left(i\right)-a\left(i\right)}{max\left(a\left(i\right),b\left(i\right)\right)}$$

Where (a(i)) is the average distance from the sample to other samples in the same cluster, and (b(i)) is the average minimum distance from the sample to samples in other clusters. The Silhouette Coefficient ranges from [-1, 1]. A coefficient close to 1 indicates good clustering, close to 0 indicates that the sample is on the boundary, and close to -1 indicates incorrect clustering.

(3) The Davies-Bouldin Index (DBI) is another metric for assessing clustering quality, focusing on the compactness and separation of clusters. The calculation formula is as follows:


$$DBI=\frac{1}{k}\sum_{i=1}^{k}\begin{array}{c} max\\ i\neq j \end{array}\frac{\sigma_{i}+\sigma_{j}}{d\left(c_{i},c_{j}\right)}$$

Where (k) is the number of clusters, σ_i_ and σ_j_ represent the average within-cluster distances of clusters (i) and (j), and (d(c_i, c_j)) is the distance between the centroids of clusters (i) and (j).

**Figure 1:**
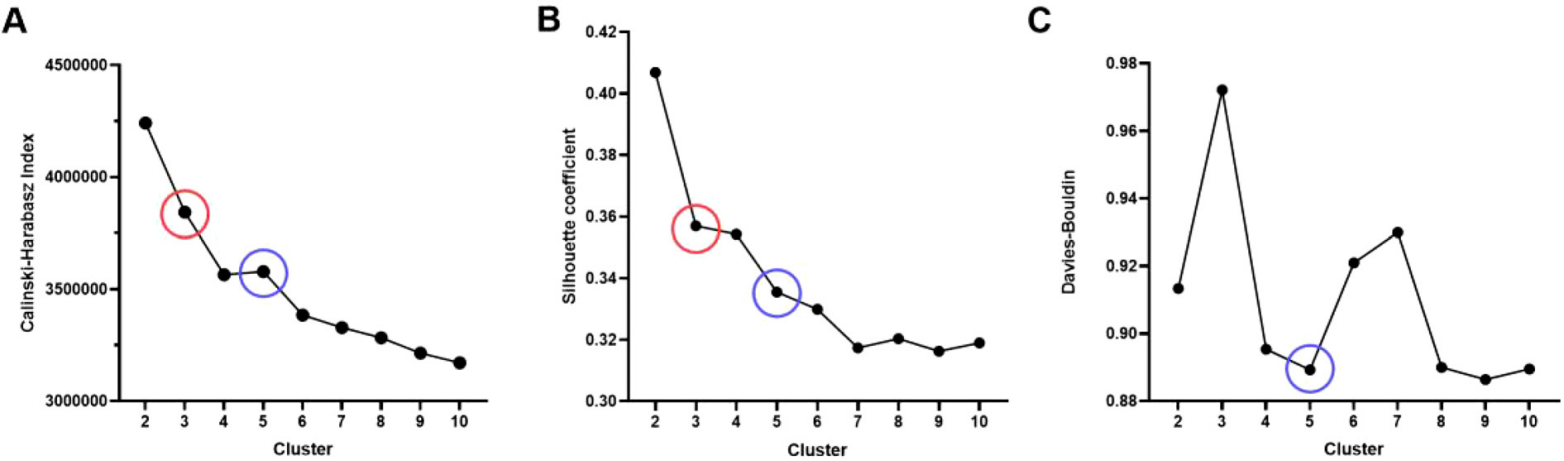
Evaluation of habitat analysis indicators. **(A)** Calinski-Harabasz index; **(B)** Silhouette Coefficient; **(C)** Davies-Bouldin Index.

In summary, considering the Calinski-Harabasz index, Silhouette Coefficient, and Davies-Bouldin Index, the clustering scheme with (K=5) is the most ideal, providing a good balance between compactness and clustering separation. This ultimately segments the tumor into five habitat regions: Tumor Habitat 1 (Habitat 1, H1), Habitat 2 (H2), Habitat 3 (H3), Habitat 4 (H4), and Habitat 5 (H5), as detailed in **Figure 1**.

### 2.6 MRI feature extraction

We used the PyRadiomics package in Python 3.10 to effectively extract radiomic features from tumor regions in MRI images. This package allows us to extract radiomic features from three sub-regions in three sequences: T1-weighted contrast-enhanced imaging, fluid-attenuated inversion recovery, and fractional anisotropy, resulting in nine total regions. These features can be classified into three categories: geometric shape, intensity distribution, and texture patterns. Geometric shape features describe the tumor’s three-dimensional morphology, intensity features represent the first-order statistical distribution of voxel intensities within the tumor, and texture features characterize intensity patterns, including second-order and higher-order spatial distributions.

To extract texture features, we utilized several methods: the Gray Level Co-occurrence Matrix (GLCM), Gray Level Run Length Matrix (GLRLM), Gray Level Size Zone Matrix (GLSZM), Gray Level Dependence Matrix (GLDM), and Neighborhood Gray Tone Difference Matrix (NGTDM), each designed to capture different aspects of image texture. In each patient’s MRI single sequence, a total of 2,153 features will be extracted from each region, comprising 14 geometric shape features, 18 first-order intensity features, 75 texture features, and additional first-order and texture features derived from filtering transformations.

These features are utilized to quantify various dimensions of the tumor, thereby revealing its characteristics and properties. For a detailed introduction to the features, please refer to the official PyRadiomics website (https://pyradiomics.readthedocs.io/en/latest/features.html).

### 2.7 Radiomic feature selection, model construction, and statistical analysis

**Figure 2:**
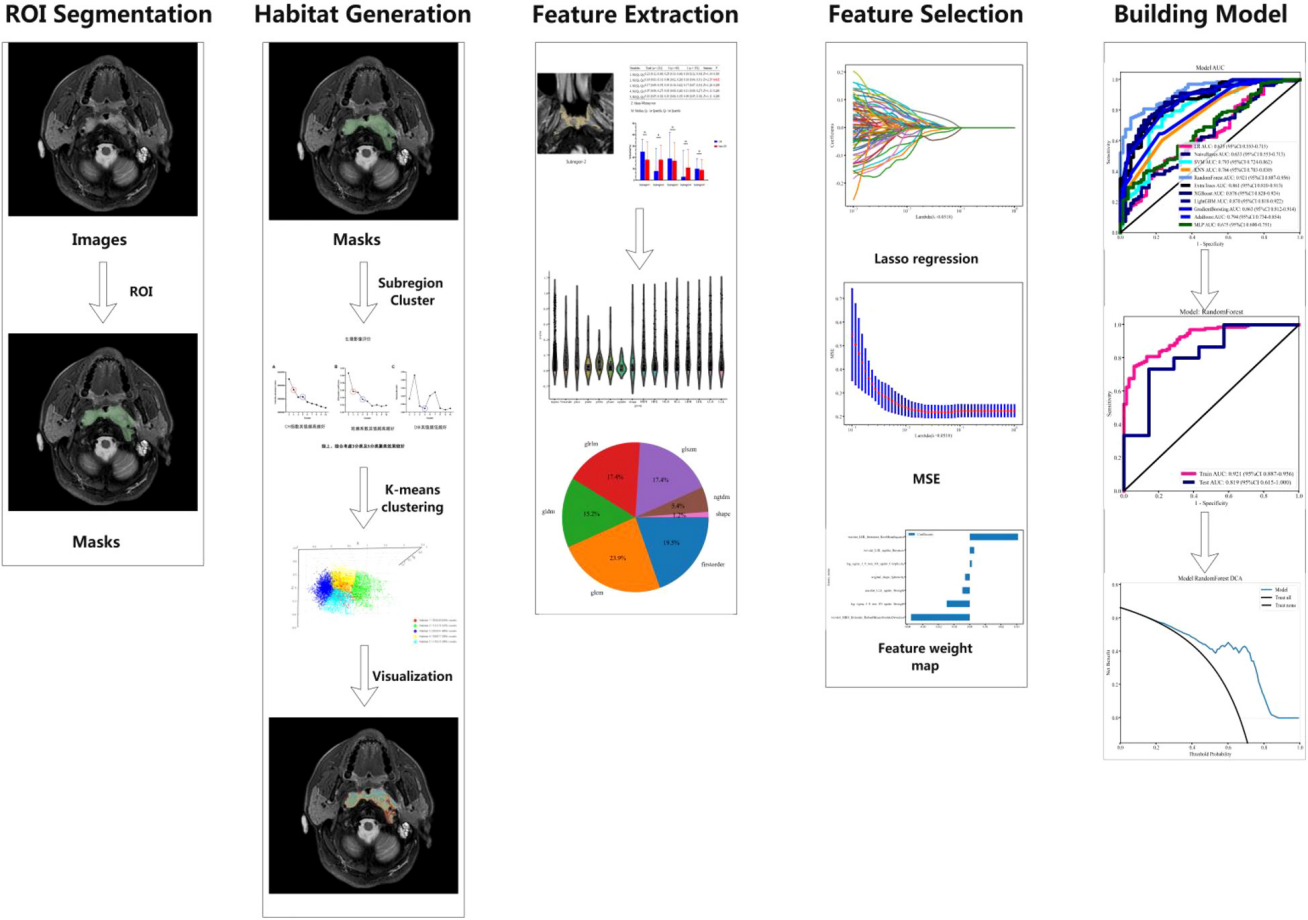
Workflow diagram of this study.

Patients were randomly divided into a training set and a testing set in a 7:3 ratio. The consistency of observers, both between and within them, was measured using the ICC. Radiomic features were considered stable if the ICC exceeded 0.75. Feature selection was conducted using two methods: Maximum Relevance Minimum Redundancy (mRMR) and Least Absolute Shrinkage and Selection Operator (LASSO). To prevent overfitting, the feature selection process utilized 10-fold cross-validation along with parameter tuning. Comparisons of categorical variables between groups were conducted using the chi-square test (χ² test). The discriminative ability of all models in the training and testing sets was evaluated using the area under the ROC curve (AUC), sensitivity, specificity, positive predictive value, and negative predictive value. The AUC values of all models in the training and testing sets were compared using the DeLong test. The calibration performance of the models was assessed using the Hosmer-Lemeshow (H-L) test, and the clinical utility of the models was evaluated using clinical decision curves. A *P*-value of less than 0.05 was considered statistically significant (**Figure 2**).

## 3 Results

### 3.1 Clinical baseline characteristics of LA-NPC patients

Among the 265 enrolled patients with LA-NPC, 90 patients (40.0%) were classified in the CR group, while 175 patients (60.0%) were classified in the non-CR group. Random assignment for the training and testing sets showed no significant differences in clinical factors between the two groups (*P* > 0.05 for all) (**Table 1**).

**Table 1:** General Information and Clinicopathological Characteristics of Patients in the Training and Testing Cohort.

Variables	Training Set - Overview (n=184)	Training Set - CR Group (n=56)	Training Set - Non-CR Group (n=128)	*P* Value	Testing Set - Overview (n=81)	Testing Set - CR Group (n=34)	Testing Set-Non-CR Group (n=47)	*P* Value
Age	42.60 ± 10.21	42.55 ± 9.03	42.62 ± 10.72	0.965	43.54 ± 11.00	43.62 ± 9.75	43.49 ± 11.93	0.959
Gender				0.095				0.272
male	141 (76.63)	38 (67.86)	103 (80.47)		61 (75.31)	23 (67.65)	38 (80.85)	
female	43 (23.37)	18 (32.14)	25 (19.53)		20 (24.69)	11 (32.35)	9 (19.15)	
Clinical Stage				0.332				0.5
III	53 (28.80)	18 (32.1)	35 (27.34)		23 (28.40)	11 (32.35)	12 (25.53)	
IVa	131 (71.20)	38 (67.90)	93 (72.66)		58 (71.60)	23 (67.65)	35 (74.47)	
Body Mass Index	22.33 ± 3.25	21.62 ± 2.85	22.65 ± 3.37	0.048	22.76 ± 3.07	22.55 ± 2.67	22.91 ± 3.35	0.6
WHO Classification				0.095				0.463
Differentiated non-keratinous carcinoma	14 (7.61)	1 (1.79)	13 (10.16)		11 (13.58)	3 (8.82)	8 (17.02)	
Undifferentiated non-keratinous carcinoma	170 (92.39)	55 (98.21)	115 (89.84)		70 (86.42)	31 (91.18)	39 (82.98)	
EBV-DNA (IU/ml) (%)				0.506				0.691
<5000	120 (65.22	39 (69.64)	81 (63.28)		54 (66.67)	24 (70.59)	30 (63.83)	
≥5000	64 (34.78)	17 (30.36)	47 (36.72)		27 (33.33)	10 (29.41)	17 (36.17)	
White Blood Cell Count (×10^9^/L)	7.35 ± 1.97	7.32 ± 2.34	7.37 ± 1.80	0.575	6.76 ± 1.66	6.90 ± 2.00	6.65 ± 1.37	0.969
Hemoglobin (g/L)	136.43 ± 16.17	133.50 ± 19.24	137.72 ± 14.52	0.157	138.17 ± 14.76	135.41 ± 17.11	140.17 ± 12.62	0.279
Platelet Count (×10^9^/L)	284.60 ± 72.11	288.20 ± 67.13	283.02 ± 74.39	0.741	278.33 ± 75.57	274.21 ± 78.87	281.32 ± 73.81	0.679
NE	5.31 ± 1.60	5.32 ± 1.86	5.31 ± 1.47	0.968	4.96 ± 1.47	5.36 ± 1.49	4.67 ± 1.39	0.036
Lymphocyte Ratio	2.29 ± 0.93	2.16 ± 0.75	2.35 ± 0.99	0.344	2.26 ± 0.95	2.27 ± 0.94	2.24 ± 0.97	0.863
Albumin	39.11 ± 3.39	38.33 ± 3.75	39.46 ± 3.17	0.02	39.49 ± 2.85	39.24 ± 2.58	39.67 ± 3.05	0.51
Family History				0.689				1.0
No	153 (83.15	48 (85.71)	105 (82.03		72 (88.89)	30 (88.24)	42 (89.36)	
Yes	31 (16.85)	8 (14.29)	23 (17.97)		9 (11.11)	4 (11.76)	5 (10.64)	
Smoking History				0.55				0.331
No	104 (56.52)	34 (60.71)	70 (54.69		56 (69.14	26 (76.47)	30 (63.83	
Yes	80 (43.48)	22 (39.29)	58 (45.31)		25 (30.86)	8 (23.53)	17 (36.17)	
T Stage				0.075				0.167
T1	7 (3.80)	2 (3.57)	5 (3.91)		4 (4.94)	2 (5.88)	2 (4.26)	
T2	63 (34.24	22 (39.29)	41 (32.03		27 (33.33)	12 (35.29)	15 (31.91)	
T3	64 (34.78)	12 (21.43)	52 (40.62)		28 (34.57)	15 (44.12)	13 (27.66)	
T4	50 (27.17)	20 (35.71)	30 (23.44)		22 (27.16)	5 (14.71)	17 (36.17)	
N Stage				0.728				0.706
N1	18 (9.78)	6 (10.71)	12 (9.38)		12 (14.81)	5 (14.71)	7 (14.89)	
N2	79 (42.93)	26 (46.43)	53 (41.41)		35 (43.21)	13 (38.24)	22 (46.81)	
N3	87 (47.28)	24 (42.86)	63 (49.22)		34 (41.98)	16 (47.06)	18 (38.30)	

### 3.2 Establishment and efficacy of the radiomic model for habitat analysis

#### 3.2.1 Habitat sub-region analysis

**Figure 3:**
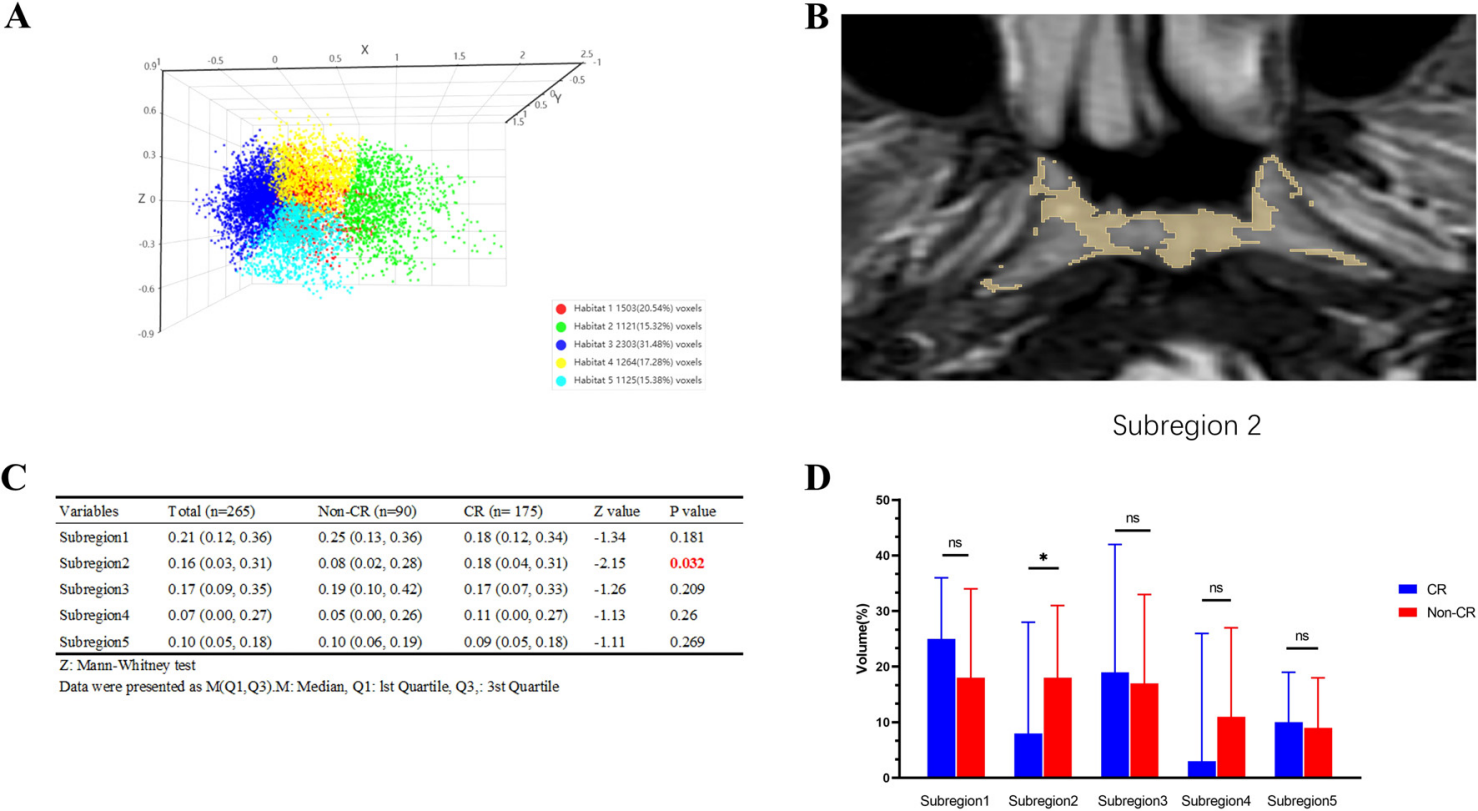
Example of habitat analysis of nasopharyngeal carcinoma lesions in subregion 2 and results of intergroup comparisons. **(A)** The Calinski-Harabasz score is defined as the ratio of the sum of squared inter-cluster distances to the sum of squared intra-cluster distances across all clusters; **(B)** Example of subregion 2; **(C)** Comparison Table of CR and Non-CR Groups; **(D)** Bar graph comparing the CR and Non-CR groups. *p < 0.05, ns: p > 0.05.

**Figure 3** presents an example of habitat analysis from the second sub-region of a NPC lesion. It highlights the statistical differences in partition volumes between the CR and non-CR groups. The results show a significant difference in volume distribution in habitat sub-region 2 between the CR and non-CR groups (*P* = 0.032). This suggests that the volumetric characteristics of specific habitat sub-regions are linked to early treatment responses.

#### 3.2.2 Related omics features

**Figure 4:**
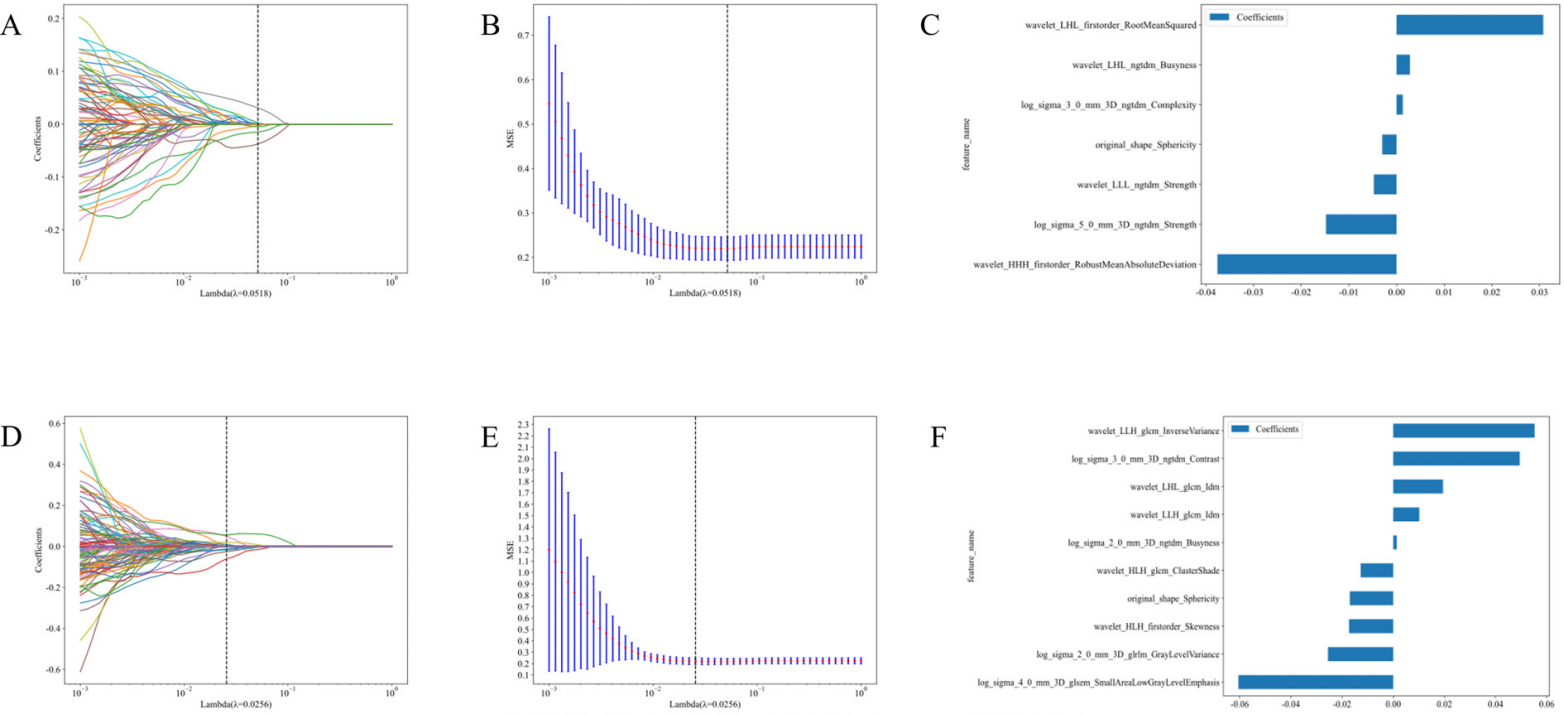
Feature selection and feature importance distribution map of the second habitat region in the CE-T1WI and T2-FS sequences. **(A)** Cross-validation in CE-T1WI; **(B)** Lasso regression path diagram IN CE-T1WI.; **(C)** Feature Importance in CE-T1WI; **(D)** Cross-validation in T2-FS; **(E)** Lasso regression path diagram in T2-FS; **(F)** Feature Importance in T2-FS.

Based on the habitat sub-region analysis results, imaging features from the sub-region 2 were extracted. Following Spearman correlation analysis and Lasso dimensionality reduction, optimal features for each group were identified, resulting in 7 features from the CE-T1WI sequence and 10 features from the T2-FS sequence. Detailed information regarding these features is present ed in **Figure 4**.

#### 3.2.3 Predictive performance of different models

This study created two radiomic models using the selected optimal features: the CE-T1WI sequence habitat model and the T2-FS sequence habitat model. We conducted a comprehensive comparison of various machine learning models, assessing their performance on training and testing sets using metrics like accuracy, AUC, sensitivity, specificity, and precision.

In evaluating the CE-T1WI sequence habitat model, Extra Trees, Random Forest (RF), and LightGBM showed balanced and robust performance. They achieved high AUC scores while maintaining consistent sensitivity and specificity across both the training and testing sets. These models exhibited strong generalization capabilities with no apparent overfitting, rendering them suitable for practical applications. Conversely, the k-nearest neighbors (KNN) model displayed significant discrepancies between training and testing results, indicating poor generalization and potential overfitting. Logistic Regression and Naive Bayes exhibited notable variations in training and testing metrics but maintained good specificity, making them appropriate for scenarios with high false positive requirements.

**Figure 5:**
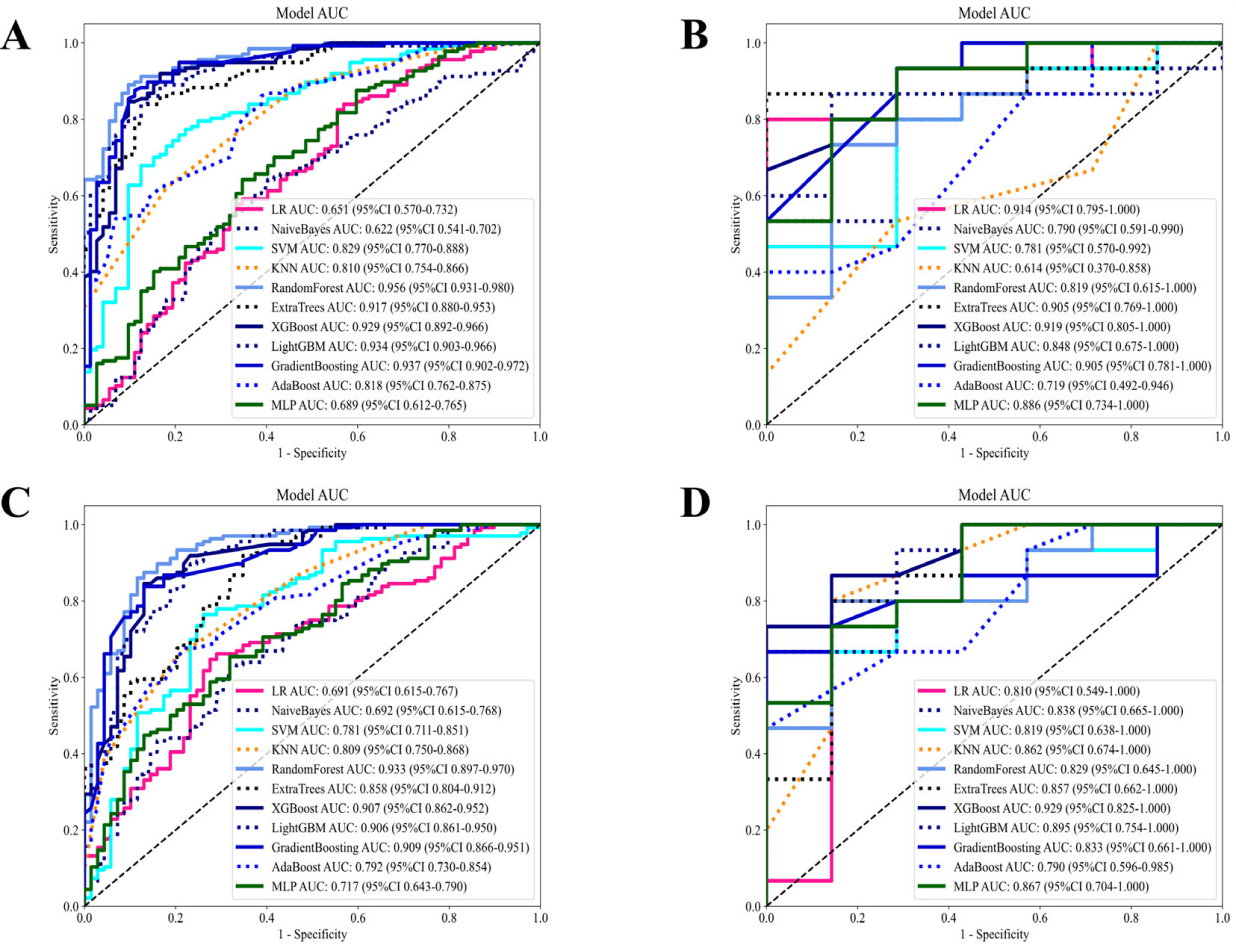
Diagnostic performance of different models based on habitat radiomics in the CE-T1WI and T2-FS sequences. **(A)** Diagnostic performance of different machine models in the CE-T1WI habitat radiomics training set; **(B)** Diagnostic performance of different machine models in the CE-T1WI habitat radiomics test set; **(C)** Diagnostic performance of different machine models in the T2-FS habitat radiomics training set; **(D)** Diagnostic performance of different machine models in the T2-FS habitat radiomics test set.

After evaluating the diagnostic performance of all machine learning models, we selected RF as the final deployment model, achieving AUC scores of 0.921 on the training set and 0.819 on the testing set. This model demonstrated strong classification ability while maintaining a balance between sensitivity and specificity. Although Extra Trees and LightGBM performed well, RF exhibited a slight advantage in stability and consistency. Consequently, RF was selected as the final deployment model for the CE-T1WI sequence habitat model, ensuring reliable results in practical applications due to its optimal balance between diagnostic performance and generalization capability (**Figures 5A, B**; **Table S1**).

In the T2-FS sequence habitat model, RF distinguished itself in the comparison of diagnostic performance among all models and was selected as the final deployment model. This model achieved AUC scores of 0.933 and 0.829 on the training and testing sets, respectively, demonstrating strong discrimination ability while maintaining balance in sensitivity and specificity metrics. Although Extra Trees, XGBoost, and Gradient Boosting performed well, RF had a slight advantage in the stability and consistency of evaluation metrics. Thus, we selected RF as the final deployment model for the T2-FS sequence habitat model, ensuring reliable results in practical applications due to its optimal balance between diagnostic performance and generalization capability (**Figures 5C, D**; **Table S2**).

#### 3.2.4 Establishment of the optimal habitat model

**Figure 6:**
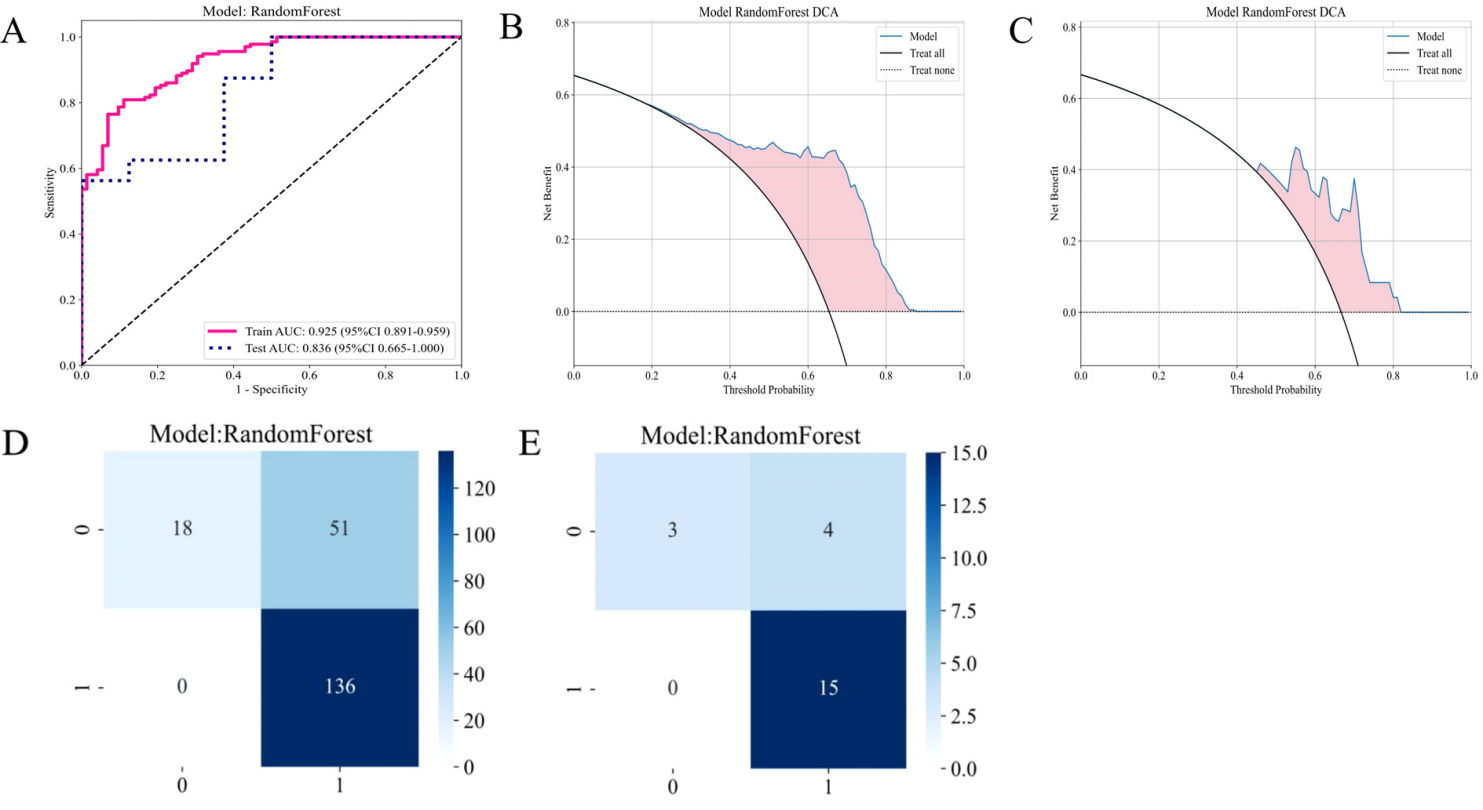
Diagnostic performance of the habitat-based RF model constructed using the CE-T1WI sequence. **(A)** ROC curves for the training and test sets; **(B)** DCA curve for the training set; **(C)** DCA curve for the test set; **(D)** Confusion matrix for the training set; **(E)** Confusion matrix for the test set.

**Figure 6A** presents the ROC curves for the training and testing sets, assessing the classification performance of the RF model on both datasets. The AUC for the training set was 0.925, while the AUC for the testing set was 0.836, indicating satisfactory performance of the model on the test set. **Figures 6B, C** show the decision curve analyses for the training and test sets. These analyses evaluate the model’s clinical net benefit at different thresholds. These analyses help determine whether the model is superior to treating or not treating all patients at different treatment thresholds. The blue curve in the decision curve represents the model’s net benefit; if it exceeds the “treat all patients” or “do not treat patients” curves, it indicates that the model performs better in practical applications at the corresponding threshold. These curves evaluate the practical application value of the model, particularly in developing personalized treatment plans in clinical settings.

**Figure 7:**
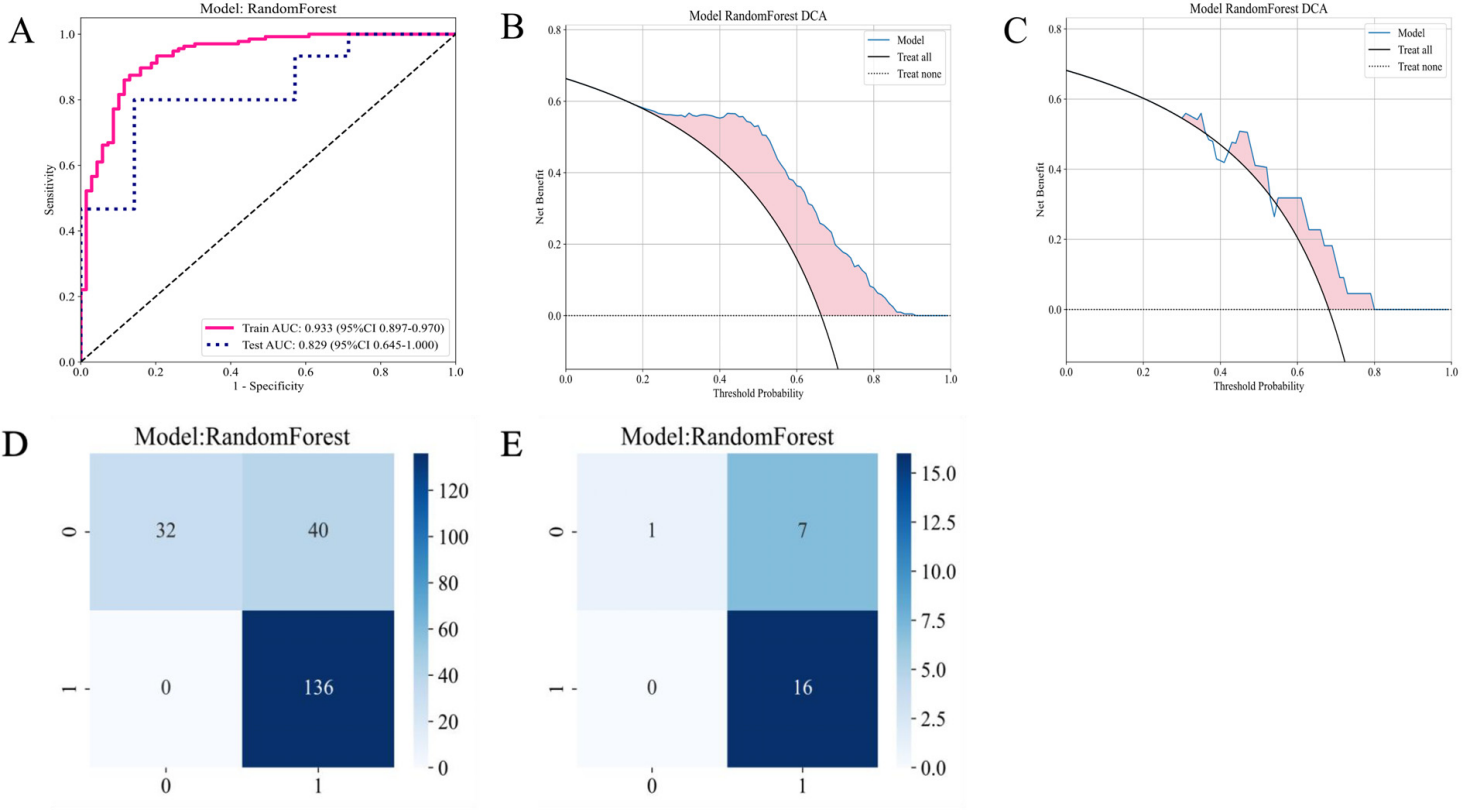
Diagnostic performance of the habitat-based RF model constructed using the T2-FS sequence. **(A)** ROC curves for the training and test sets; **(B)** DCA curve for the training set; **(C)** DCA curve for the test set; **(D)** Confusion matrix for the training set; **(E)** Confusion matrix for the test set.

**Figure 7A** presents the ROC curve of the RF model on the T2-FS sequence. The AUC for the training set was 0.933, and the AUC for the test set was 0.829, indicating that the model’s performance on the T2WI sequence is similar to that of CE-T1WI, suggesting that both imaging sequences possess comparable predictive capabilities for patient responses. The DCA plots in **Figures 7B, C** demonstrate that the RF model based on the T2-FS sequence continues to provide a net benefit across different threshold ranges, particularly showing significant benefits at lower thresholds. Compared to the CE-T1WI sequence, the DCA curve of T2-FS exhibits a similar trend, implying that models for both sequences have substantial potential clinical application value.

## 4 Discussion

Emerging evidence substantiates the critical role of tumor habitat heterogeneity in cancer pathophysiology and therapeutic resistance, with multiple studies demonstrating spatial correlations between microenvironmental features and clinical outcomes (27–29). This body of research particularly highlights how distinct habitat subdomains contribute to treatment refractoriness, potentially accounting for suboptimal therapeutic responses and adverse prognoses. Building upon these foundations, we developed a multiparametric MRI-based radiomic habitat segmentation model to predict early therapeutic responses in LA-NPC patients undergoing ICT. Our methodology employed habitat segmentation analysis to quantify tumor spatial heterogeneity, revealing statistically significant associations between habitat-specific volumetric profiles and treatment outcomes. Notably, habitat subdomain 2 exhibited significant volumetric disparities between CR and non-CR cohorts (*P*=0.032), suggesting microenvironment-driven chemotherapeutic resistance mechanisms. Three plausible pathways explain this spatial efficacy variation: (1) Differential vascular permeability establishing intratumoral drug concentration gradients (30), (2) Regional variations in tumor proliferation kinetics modulating chemosensitivity (31), and (3) Stromal compartmentalization creating fibrotic sanctuaries that impede drug penetration (32). Through a meticulous feature selection process, we extracted 7 and 10 optimal features based on the CE-T1WI and T2-FS sequences, respectively. The constructed RF models exhibited high AUC values on both the training and test sets, with CE-T1WI achieving values of 0.921 and 0.819, and T2-FS reaching 0.933 and 0.819. These impressive results emphasize the potential of radiomic methods in accurately predicting tumor responses, thereby paving the way for more personalized and effective treatment strategies in oncology.

Our comprehensive comparison of 11 machine learning algorithms revealed critical performance differences (**Tables S1, S2**). While gradient boosting demonstrated competitive AUCs (CE-T1WI: 0.863 training/0.905 testing; T2-FS: 0.909/0.833), it exhibited greater variance between sequences. Similarly, SVM showed sequence-dependent instability (CE-T1WI AUC 0.793 vs. T2-FS 0.781 in training). The RF’s superior stability likely stems from its inherent ensemble design - bootstrap aggregating reduces variance while feature subspace sampling minimizes overfitting. This dual randomization mechanism proved particularly effective in handling our high-dimensional radiomic features while maintaining generalizability across MRI sequences. Moreover, compared to single-tree models like XGBoost (test set AUC 0.919 vs. 0.929 for CE-T1WI vs. T2-FS), the forest architecture better accommodated heterogeneous response patterns through multi-tree consensus (33).

Previous studies have successfully utilized traditional radiomic models to predict treatment outcomes in patients with NPC (12, 34). However, these studies did not thoroughly investigate intratumoral heterogeneity. Given the significant presence of tumor heterogeneity in NPC (35), we conducted a comprehensive habitat sub-region analysis to develop predictive models for early treatment responses. Habitat analysis, as an emerging radiomic approach, has garnered increasing attention in recent years for its potential in tumor evaluation. By deeply analyzing the tumor microenvironment, habitat analysis can reveal tumor heterogeneity and its relationship with clinical characteristics. For instance, studies have demonstrated that volumetric features of habitat sub-regions are closely associated with patient prognosis, providing critical evidence for personalized treatment. Additionally, habitat analysis enables the quantification of biological properties across different tumor regions, aiding clinicians in better understanding tumor behavior and optimizing therapeutic strategies. Habitat sub-region analysis captures multidimensional tumor information, including texture, intensity, biological characteristics, and tumor behavior (13). To date, habitat sub-region analysis has shown promising predictive value across various cancer types (21, 36, 37). Moreover, a study by Xu explored the application of habitat analysis in predicting NPC prognosis, establishing a risk scoring model based on PET-CT images, further demonstrating the clinical utility of habitat analysis (22). In clinical diagnosis and treatment, MRI is regarded as the gold standard imaging modality. Therefore, in this study, we delineated sub-regions based on MRI images and incorporated machine learning algorithms into the modeling process. Consistent with previous findings, our results indicate that the habitat sub-region model holds significant importance in predicting NPC prognosis. Some studies have subdivided regions of interest into intra-tumoral, peri-tumoral, tumor core, or surrounding edema regions for habitat analysis (14, 38). These subdivisions are specifically designed based on the unique characteristics of the cancer under investigation. Our approach, through detailed segmentation of tumor habitats, offers new perspectives on understanding tumor biology and therapeutic responses. This aligns with current research trends and underscores the importance of personalized treatment. From a clinical perspective, habitat sub-region analysis provides a novel entry point for individualized treatment. For patients with larger sub-region 2 volumes, even when their clinical staging is similar to those with favorable responses, intratumoral heterogeneity may result in suboptimal ICT efficacy. In such cases, clinicians may consider adjusting chemotherapy regimens (e.g., increasing drug dosage or combining targeted therapies) or planning adjuvant treatment strategies in advance. Additionally, dynamic monitoring of habitat sub-regions may serve as an important complement to treatment evaluation. For example, during ICT, continuous MRI scans to track volumetric changes in specific sub-regions could enable early identification of treatment resistance, allowing timely intervention.

Habitat sub-region analysis has demonstrated predictive value across various cancers. For instance, in glioblastoma, sub-regions with active angiogenesis are significantly associated with shorter patient survival (17); in lung cancer, heterogeneous sub-regions at tumor margins have been shown to correlate with increased metastatic risk (15). This study is the first to apply this approach to predicting chemotherapy responses in NPC, further validating its cross-cancer applicability. However, unlike other cancers, NPC’s unique microenvironment, influenced by Epstein-Barr virus (EBV) infection, may endow it with distinct characteristics (4). Future studies should explore the molecular mechanisms of habitat sub-regions in conjunction with virological markers, such as EBV-DNA load.

While our findings have significant implications, we must also recognize several important limitations. Firstly, our sample size of 265 cases is statistically representative. However, its relatively small size may limit the generalizability of the results. To strengthen future studies, researchers should expand the sample size to include a broader and more diverse patient population, which would provide more comprehensive insights. Secondly, it is important to recognize that this study is a retrospective analysis, which carries a risk of selection bias that may skew the results. Therefore, conducting prospective studies will be essential to validate our findings and to further explore the applicability of habitat analysis across different types of cancer, ensuring that the conclusions drawn are both reliable and applicable.

Future research should explore key areas to expand on our findings. First, analyzing how different characteristics of habitat sub-regions affect treatment responses could inform the development of tailored therapies for individual patients. Second, exploring comprehensive analyses that integrate multimodal imaging techniques, such as PET/CT and MRI, could significantly enhance predictive accuracy, allowing for more precise assessments of treatment efficacy. Additionally, applying this model in clinical trials to assess its practical utility in personalized treatment approaches will be an important next step in translating our research into clinical practice.

In summary, this study highlights the remarkable potential of radiomics in predicting the response of patients with LA-NPC to ICT. The insights gained from this research not only provide a solid foundation for the development of personalized treatment strategies but also offer valuable guidance for future studies that aim to further investigate the intricate relationship between tumor microenvironments and treatment outcomes.

## Data Availability

The original contributions presented in the study are included in the article/**Supplementary Material**. Further inquiries can be directed to the corresponding author.

## References

[1] Guo Z Bao MH Fan YX Zhang Y Liu HY Zhou XL. Genetic polymorphisms of long non-coding RNA linc00312 are associated with susceptibility and predict poor survival of nasopharyngeal carcinoma. Front Cell Dev Biol. (2021) 9:698558. doi: 10.3389/fcell.2021.698558 34336850 PMC8322760

[2] Renaud S Lefebvre A Mordon S Moralès O Delhem N. Novel therapies boosting T cell immunity in epstein barr virus-associated nasopharyngeal carcinoma. Int J Mol Sci. (2020) 21:4292. doi: 10.3390/ijms21124292 32560253 PMC7352617

[3] Sung H Ferlay J Siegel RL Laversanne M Soerjomataram I Jemal A. Global cancer statistics 2020: GLOBOCAN estimates of incidence and mortality worldwide for 36 cancers in 185 countries. CA Cancer J Clin. (2021) 71:209–49. doi: 10.3322/caac.21660 33538338

[4] Chen Y-P Chan ATC Le Q-T Blanchard P Sun Y Ma J. Nasopharyngeal carcinoma. Lancet Lond Engl. (2019) 394:64–80. doi: 10.1016/S0140-6736(19)30956-0 31178151

[5] Marks JE Phillips JL Menck HR. The National Cancer Data Base report on the relationship of race and national origin to the histology of nasopharyngeal carcinoma. Cancer. (1998) 83:582–8. doi: 10.1002/(SICI)1097-0142(19980801)83:3<582::AID-CNCR29>3.0.CO;2-R 9690553

[6] Chen Y-P Tang L-L Yang Q Poh S-S Hui EP Chan ATC. Induction chemotherapy plus concurrent chemoradiotherapy in endemic nasopharyngeal carcinoma: individual patient data pooled analysis of four randomized trials. Clin Cancer Res Off J Am Assoc Cancer Res. (2018) 24:1824–33. doi: 10.1158/1078-0432.CCR-17-2656 29431618

[7] Liu S-L Sun X-S Yan J-J Chen Q-Y Lin H-X Wen Y-F. Optimal cumulative cisplatin dose in nasopharyngeal carcinoma patients based on induction chemotherapy response. Radiother Oncol J Eur Soc Ther Radiol Oncol. (2019) 137:83–94. doi: 10.1016/j.radonc.2019.04.020 31078941

[8] Xiao-ping Y Jing H Fei-ping L Yin H Qiang L Lanlan W. Intravoxel incoherent motion MRI for predicting early response to induction chemotherapy and chemoradiotherapy in patients with nasopharyngeal carcinoma. J Magn Reson Imaging JMRI. (2016) 43:1179–90. doi: 10.1002/jmri.25075 26540374

[9] Zhao D-W Fan W-J Meng L-L Luo Y-R Wei J Liu K. Comparison of the pre-treatment functional MRI metrics’ efficacy in predicting Locoregionally advanced nasopharyngeal carcinoma response to induction chemotherapy. Cancer Imaging Off Publ Int Cancer Imaging Soc. (2021) 21:59. doi: 10.1186/s40644-021-00428-0 PMC8579637 34758876

[10] Li Y Xu W Fei Y Wu M Yuan J Qiu L. A MRI-based radiomics model for predicting the response to anlotinb combined with temozolomide in recurrent Malignant glioma patients. Discovery Oncol. (2023) 14:154. doi: 10.1007/s12672-023-00751-x PMC10447352 37612579

[11] Gillies RJ Kinahan PE Hricak H. Radiomics: images are more than pictures, they are data. Radiology. (2016) 278:563–77. doi: 10.1148/radiol.2015151169 PMC4734157 26579733

[12] Aerts HJWL. The potential of radiomic-based phenotyping in precision medicine: A review. JAMA Oncol. (2016) 2:1636. doi: 10.1001/jamaoncol.2016.2631 27541161

[13] Gatenby RA Grove O Gillies RJ. Quantitative imaging in cancer evolution and ecology. Radiology. (2013) 269:8–14. doi: 10.1148/radiol.13122697 24062559 PMC3781355

[14] Park JE Kim HS Kim N Park SY Kim Y-H Kim JH. Spatiotemporal heterogeneity in multiparametric physiologic MRI is associated with patient outcomes in IDH-wildtype glioblastoma. Clin Cancer Res. (2021) 27:237–45. doi: 10.1158/1078-0432.CCR-20-2156 33028594

[15] Kim J Ryu S-Y Lee S-H Lee HY Park H. Clustering approach to identify intratumour heterogeneity combining FDG PET and diffusion-weighted MRI in lung adenocarcinoma. Eur Radiol. (2019) 29:468–75. doi: 10.1007/s00330-018-5590-0 29922931

[16] Wu J Cao G Sun X Lee J Rubin DL Napel S. Intratumoral spatial heterogeneity at perfusion MR imaging predicts recurrence-free survival in locally advanced breast cancer treated with neoadjuvant chemotherapy. Radiology. (2018) 288:26–35. doi: 10.1148/radiol.2018172462 29714680 PMC6029132

[17] Cui Y Tha KK Terasaka S Yamaguchi S Wang J Kudo K. Prognostic imaging biomarkers in glioblastoma: development and independent validation on the basis of multiregion and quantitative analysis of MR images. Radiology. (2016) 278:546–53. doi: 10.1148/radiol.2015150358 PMC4734164 26348233

[18] Juan-Albarracín J Fuster-Garcia E Pérez-Girbés A Aparici-Robles F Alberich-Bayarri Á Revert-Ventura A. Glioblastoma: vascular habitats detected at preoperative dynamic susceptibility-weighted contrast-enhanced perfusion MR imaging predict survival. Radiology. (2018) 287:944–54. doi: 10.1148/radiol.2017170845 29357274

[19] Beig N Bera K Prasanna P Antunes J Correa R Singh S. Radiogenomic-based survival risk stratification of tumor habitat on gd-T1w MRI is associated with biological processes in glioblastoma. Clin Cancer Res. (2020) 26:1866–76. doi: 10.1158/1078-0432.CCR-19-2556 PMC7165059 32079590

[20] Wu J Gensheimer MF Dong X Rubin DL Napel S Diehn M. Robust intratumor partitioning to identify high-risk subregions in lung cancer: A pilot study. Int J Radiat Oncol. (2016) 95:1504–12. doi: 10.1016/j.ijrobp.2016.03.018 PMC4969127 27212196

[21] Xia W Chen Y Zhang R Yan Z Zhou X Zhang B. Radiogenomics of hepatocellular carcinoma: multiregion analysis-based identification of prognostic imaging biomarkers by integrating gene data—a preliminary study. Phys Med Biol. (2018) 63:035044. doi: 10.1088/1361-6560/aaa609 29311419

[22] Xu H Lv W Feng H Du D Yuan Q Wang Q. Subregional radiomics analysis of PET/CT imaging with intratumor partitioning: application to prognosis for nasopharyngeal carcinoma. Mol Imaging Biol. (2020) 22:1414–26. doi: 10.1007/s11307-019-01439-x 31659574

[23] Yuan J Wu M Qiu L Xu W Fei Y Zhu Y. Tumor habitat-based MRI features assessing early response in locally advanced nasopharyngeal carcinoma. Oral Oncol. (2024) 158:106980. doi: 10.1016/j.oraloncology.2024.106980 39151333

[24] Posner MR Hershock DM Blajman CR Mickiewicz E Winquist E Gorbounova V. Cisplatin and fluorouracil alone or with docetaxel in head and neck cancer. N Engl J Med. (2007) 357:1705–15. doi: 10.1056/NEJMoa070956 17960013

[25] Pfister DG Spencer S Adelstein D Adkins D Anzai Y Brizel DM. Head and neck cancers, version 2.2020, NCCN clinical practice guidelines in oncology. J Natl Compr Cancer Netw JNCCN. (2020) 18:873–98. doi: 10.6004/jnccn.2020.0031 32634781

[26] Eisenhauer EA Therasse P Bogaerts J Schwartz LH Sargent D Ford R. New response evaluation criteria in solid tumours: Revised RECIST guideline (version 1.1). Eur J Cancer. (2009) 45:228–47. doi: 10.1016/j.ejca.2008.10.026 19097774

[27] Li S Dai Y Chen J Yan F Yang Y. MRI-based habitat imaging in cancer treatment: current technology, applications, and challenges. Cancer Imaging Off Publ Int Cancer Imaging Soc. (2024) 24:107. doi: 10.1186/s40644-024-00758-9 PMC11328409 39148139

[28] Lee DH Park JE Kim N Park SY Kim Y-H Cho YH. Tumor habitat analysis using longitudinal physiological MRI to predict tumor recurrence after stereotactic radiosurgery for brain metastasis. Korean J Radiol. (2023) 24:235–46. doi: 10.3348/kjr.2022.0492 PMC9971843 36788768

[29] Jeong SY Park JE Kim N Kim HS. Hypovascular cellular tumor in primary central nervous system lymphoma is associated with treatment resistance: tumor habitat analysis using physiologic MRI. AJNR Am J Neuroradiol. (2022) 43:40–7. doi: 10.3174/ajnr.A7351 PMC8757556 34824097

[30] Jain RK. Normalization of tumor vasculature: an emerging concept in antiangiogenic therapy. Science. (2005) 307:58–62. doi: 10.1126/science.1104819 15637262

[31] Almendro V Marusyk A Polyak K. Cellular heterogeneity and molecular evolution in cancer. Annu Rev Pathol. (2013) 8:277–302. doi: 10.1146/annurev-pathol-020712-163923 23092187

[32] Özdemir BC Pentcheva-Hoang T Carstens JL Zheng X Wu C-C Simpson TR. Depletion of carcinoma-associated fibroblasts and fibrosis induces immunosuppression and accelerates pancreas cancer with reduced survival. Cancer Cell. (2014) 25:719–34. doi: 10.1016/j.ccr.2014.04.005 PMC4180632 24856586

[33] Becker T Rousseau A-J Geubbelmans M Burzykowski T Valkenborg D. Decision trees and random forests. Am J Orthod Dentofac Orthop Off Publ Am Assoc Orthod Its Const Soc Am Board Orthod. (2023) 164:894–7. doi: 10.1016/j.ajodo.2023.09.011 38008491

[34] Zhao L Gong J Xi Y Xu M Li C Kang X. MRI-based radiomics nomogram may predict the response to induction chemotherapy and survival in locally advanced nasopharyngeal carcinoma. Eur Radiol. (2020) 30:537–46. doi: 10.1007/s00330-019-06211-x 31372781

[35] Lin M Zhang X-L You R Liu Y-P Cai H-M Liu L-Z. Evolutionary route of nasopharyngeal carcinoma metastasis and its clinical significance. Nat Commun. (2023) 14:610. doi: 10.1038/s41467-023-35995-2 36739462 PMC9899247

[36] Tan HQ Cai J Tay SH Sim AYL Huang L Chua MLK. Cluster-based radiomics reveal spatial heterogeneity of bevacizumab response for treatment of radiotherapy-induced cerebral necrosis. Comput Struct Biotechnol J. (2024) 23:43–51. doi: 10.1016/j.csbj.2023.11.040 38125298 PMC10730953

[37] Xie C Yang P Zhang X Xu L Wang X Li X. Sub-region based radiomics analysis for survival prediction in oesophageal tumours treated by definitive concurrent chemoradiotherapy. eBioMedicine. (2019) 44:289–97. doi: 10.1016/j.ebiom.2019.05.023 PMC6606893 31129097

[38] Yang Y Han Y Zhao S Xiao G Guo L Zhang X. Spatial heterogeneity of edema region uncovers survival-relevant habitat of Glioblastoma. Eur J Radiol. (2022) 154:110423. doi: 10.1016/j.ejrad.2022.110423 35777079

