# Supplementary material for: Prediction of induction chemotherapy efficacy in patients with locally advanced nasopharyngeal carcinoma using habitat subregions derived from multi-modal MRI radiomics
Source: Front Oncol. 2025 May 14;15:1539574. doi: 10.3389/fonc.2025.1539574 (PMC12116681; doi:10.3389/fonc.2025.1539574)
Supplement: Supplementary file 1 [file Table1.docx]

TableS1 Prediction performance of different models based on habitat radiomics in the CE-T1WI sequence

| Queue | Model Name | Accuracy | AUC Value | 95% CI | Sensitivity | Specificity | PPV | NPV | F1 Score |
| --- | --- | --- | --- | --- | --- | --- | --- | --- | --- |
| Training Set | LR | 0.561 | 0.635 | 0.555-0.715 | 0.463 | 0.754 | 0.787 | 0.416 | 0.583 |
| Testing Set | LR | 0.818 | 0.914 | 0.795-1.000 | 0.733 | 1.000 | 1.000 | 0.636 | 0.846 |
| Training Set | NaiveBayes | 0.527 | 0.633 | 0.553-0.713 | 0.368 | 0.841 | 0.820 | 0.403 | 0.508 |
| Testing Set | NaiveBayes | 0.636 | 0.790 | 0.591-0.990 | 0.467 | 1.000 | 1.000 | 0.467 | 0.636 |
| Training Set | SVM | 0.756 | 0.793 | 0.724-0.862 | 0.757 | 0.754 | 0.858 | 0.612 | 0.805 |
| Testing Set | SVM | 0.727 | 0.781 | 0.570-0.992 | 0.733 | 0.714 | 0.846 | 0.556 | 0.786 |
| Training Set | KNN | 0.483 | 0.766 | 0.703-0.830 | 0.221 | 1.000 | 1.000 | 0.394 | 0.361 |
| Testing Set | KNN | 0.409 | 0.614 | 0.370-0.858 | 0.133 | 1.000 | 1.000 | 0.350 | 0.235 |
| Training Set | RandomForest | 0.810 | 0.921 | 0.887-0.956 | 0.743 | 0.942 | 0.962 | 0.650 | 0.838 |
| Testing Set | RandomForest | 0.727 | 0.819 | 0.615-1.000 | 0.667 | 0.857 | 0.909 | 0.545 | 0.769 |
| Training Set | ExtraTrees | 0.756 | 0.861 | 0.810-0.913 | 0.699 | 0.870 | 0.913 | 0.594 | 0.792 |
| Testing Set | ExtraTrees | 0.864 | 0.905 | 0.769-1.000 | 0.800 | 1.000 | 1.000 | 0.700 | 0.889 |
| Training Set | XGBoost | 0.800 | 0.876 | 0.828-0.924 | 0.809 | 0.783 | 0.880 | 0.675 | 0.843 |
| Testing Set | XGBoost | 0.727 | 0.919 | 0.804-1.000 | 0.600 | 1.000 | 1.000 | 0.538 | 0.750 |
| Training Set | LightGBM | 0.815 | 0.870 | 0.818-0.922 | 0.846 | 0.754 | 0.871 | 0.712 | 0.858 |
| Testing Set | LightGBM | 0.818 | 0.848 | 0.675-1.000 | 0.800 | 0.857 | 0.923 | 0.667 | 0.857 |
| Training Set | GradientBoosting | 0.663 | 0.863 | 0.812-0.914 | 0.522 | 0.942 | 0.947 | 0.500 | 0.673 |
| Testing Set | GradientBoosting | 0.818 | 0.905 | 0.781-1.000 | 0.867 | 0.714 | 0.867 | 0.714 | 0.867 |
| Training Set | AdaBoost | 0.571 | 0.794 | 0.734-0.854 | 0.382 | 0.942 | 0.929 | 0.436 | 0.542 |
| Testing Set | AdaBoost | 0.545 | 0.719 | 0.492-0.946 | 0.333 | 1.000 | 1.000 | 0.412 | 0.500 |
| Training Set | MLP | 0.551 | 0.675 | 0.600-0.751 | 0.397 | 0.855 | 0.844 | 0.418 | 0.540 |
| Testing Set | MLP | 0.773 | 0.886 | 0.734 -1.000 | 0.733 | 0.857 | 0.917 | 0.600 | 0.815 |

TableS2 Prediction performance of different models based on habitat radiomics in the T2WI-FS sequence

| Queue | Model Name | Accuracy | AUC Value | 95% CI | Sensitivity | Specificity | PPV | NPV | F1 Score |
| --- | --- | --- | --- | --- | --- | --- | --- | --- | --- |
| Training Set | LR | 0.673 | 0.691 | 0.615-0.767 | 0.654 | 0.710 | 0.817 | 0.510 | 0.727 |
| Testing Set | LR | 0.773 | 0.810 | 0.549-1.000 | 0.733 | 0.857 | 0.917 | 0.600 | 0.815 |
| Training Set | NaiveBayes | 0.639 | 0.692 | 0.616-0.768 | 0.618 | 0.681 | 0.792 | 0.475 | 0.694 |
| Testing Set | NaiveBayes | 0.773 | 0.838 | 0.665-1.000 | 0.733 | 0.857 | 0.917 | 0.600 | 0.815 |
| Training Set | SVM | 0.751 | 0.781 | 0.711-0.8514 | 0.757 | 0.739 | 0.851 | 0.607 | 0.802 |
| Testing Set | SVM | 0.636 | 0.819 | 0.638-1.000 | 0.467 | 1.000 | 1.000 | 0.467 | 0.636 |
| Training Set | KNN | 0.580 | 0.809 | 0.750-0.868 | 0.390 | 0.957 | 0.946 | 0.443 | 0.552 |
| Testing Set | KNN | 0.591 | 0.862 | 0.674-1.000 | 0.467 | 0.857 | 0.875 | 0.429 | 0.609 |
| Training Set | RandomForest | 0.868 | 0.933 | 0.897-0.970 | 0.868 | 0.870 | 0.929 | 0.769 | 0.897 |
| Testing Set | RandomForest | 0.773 | 0.829 | 0.645-1.000 | 0.733 | 0.857 | 0.917 | 0.600 | 0.815 |
| Training Set | ExtraTrees | 0.824 | 0.858 | 0.804-0.912 | 0.912 | 0.652 | 0.838 | 0.789 | 0.873 |
| Testing Set | ExtraTrees | 0.773 | 0.857 | 0.662-1.000 | 0.733 | 0.857 | 0.917 | 0.600 | 0.815 |
| Training Set | XGBoost | 0.834 | 0.907 | 0.862-0.952 | 0.816 | 0.870 | 0.925 | 0.706 | 0.867 |
| Testing Set | XGBoost | 0.773 | 0.929 | 0.825-1.000 | 0.667 | 1.000 | 1.000 | 0.583 | 0.800 |
| Training Set | LightGBM | 0.859 | 0.906 | 0.861-0.950 | 0.919 | 0.739 | 0.874 | 0.823 | 0.896 |
| Testing Set | LightGBM | 0.727 | 0.895 | 0.754-1.000 | 0.600 | 1.000 | 1.000 | 0.538 | 0.750 |
| Training Set | GradientBoosting | 0.844 | 0.909 | 0.866-0.951 | 0.831 | 0.870 | 0.926 | 0.723 | 0.876 |
| Testing Set | GradientBoosting | 0.727 | 0.833 | 0.661-1.000 | 0.600 | 1.000 | 1.000 | 0.538 | 0.750 |
| Training Set | AdaBoost | 0.668 | 0.792 | 0.730-0.853 | 0.588 | 0.826 | 0.870 | 0.504 | 0.702 |
| Testing Set | AdaBoost | 0.545 | 0.790 | 0.596-0.985 | 0.333 | 1.000 | 1.000 | 0.412 | 0.500 |
| Training Set | MLP | 0.659 | 0.717 | 0.643-0.790 | 0.647 | 0.681 | 0.800 | 0.495 | 0.715 |
| Testing Set | MLP | 0.727 | 0.867 | 0.704-1.000 | 0.667 | 0.857 | 0.909 | 0.545 | 0.769 |
